# BE EPIC-VR: TRANSLATING AN IN-PERSON, PERSON-CENTERED COMMUNICATION TRAINING PROGRAM INTO VIRTUAL REALITY

**DOI:** 10.1093/geroni/igad104.1052

**Published:** 2023-12-21

**Authors:** Marie Savundranayagam, Allison Chen, Grace Norris, Annette Schumann, Simran Kaur, Jennifer Campos, Joseph Orange

**Affiliations:** Western University, London, Ontario, Canada; Western University - Sam Katz Community Health & Aging Research Unit, London, Ontario, Canada; Sam Katz Community Health & Aging Research Unit, London, Ontario, Canada; Western University - Sam Katz Community Health & Aging Research Unit, London, Ontario, Canada; Western University-Sam Katz Community Health & Aging Research Unit, London, Ontario, Canada; University of Toronto, Toronto, Ontario, Canada; Western University, London, Ontario, Canada

## Abstract

Be EPIC is a dementia-specific, person-centered communication training program for frontline healthcare workers that uses simulations with trained actors, reflection, and feedback. Scaling Be EPIC is limited by resource and methodology demands of recruiting and training actors and consistency of delivery across sites. Virtual reality (VR) is a promising solution to the problem of scaling Be EPIC because it provides interactive, realistic, and consistent simulations. However, there is limited research on implementing VR to train frontline healthcare workers. Guided by the Consolidated Framework for Implementation Research, the current study used an effectiveness-implementation hybrid design to compare simulation experiences of Be EPIC-VR with Be EPIC-in person and real-world clinical encounters. The study also explored factors that can influence the future implementation of Be EPIC-VR in long-term care home settings during the pre-implementation period. Six frontline healthcare workers who completed the Be EPIC in-person version with trained actors also completed the same assessment simulation in VR, followed by an interview. Thematic analyses revealed two themes that contributed to the simulation’s realism: the immersive nature of the virtual environment and the accuracy of the avatar’s visual, verbal, and behavioral characteristics. Moreover, the factors influencing the successful implementation of VR simulations in long-term care home settings include sufficient technological infrastructure and workplace personnel who can assist with Be EPIC-VR implementation. The findings highlight the potential to scale Be EPIC using VR and provides insights into the iterative process involved in translating an in-person training into a VR training program.

